# Recommending plant taxa for supporting on-site species identification

**DOI:** 10.1186/s12859-018-2201-7

**Published:** 2018-05-30

**Authors:** Hans Christian Wittich, Marco Seeland, Jana Wäldchen, Michael Rzanny, Patrick Mäder

**Affiliations:** 1 Institute for Computer and Systems Engineering, Technische Universität Ilmenau, Helmholtzplatz 5, Ilmenau, 98693 Germany; 2 Department Biogeochemical Integration, Max-Planck-Institute for Biogeochemistry, Hans-Knöll-Str. 10, Jena, 07745 Germany

**Keywords:** Plant identification, Location-based, Classification, Spatio-temporal context, Recommender system, Occurrence prediction, Plant distribution

## Abstract

**Background:**

Predicting a list of plant taxa most likely to be observed at a given geographical location and time is useful for many scenarios in biodiversity informatics. Since efficient plant species identification is impeded mainly by the large number of possible candidate species, providing a shortlist of likely candidates can help significantly expedite the task. Whereas species distribution models heavily rely on geo-referenced occurrence data, such information still remains largely unused for plant taxa identification tools.

**Results:**

In this paper, we conduct a study on the feasibility of computing a ranked shortlist of plant taxa likely to be encountered by an observer in the field. We use the territory of Germany as case study with a total of 7.62M records of freely available plant presence-absence data and occurrence records for 2.7k plant taxa. We systematically study achievable recommendation quality based on two types of source data: binary presence-absence data and individual occurrence records. Furthermore, we study strategies for aggregating records into a taxa recommendation based on location and date of an observation.

**Conclusion:**

We evaluate recommendations using 28k geo-referenced and taxa-labeled plant images hosted on the Flickr website as an independent test dataset. Relying on location information from presence-absence data alone results in an average recall of 82%. However, we find that occurrence records are complementary to presence-absence data and using both in combination yields considerably higher recall of 96% along with improved ranking metrics. Ultimately, by reducing the list of candidate taxa by an average of 62%, a spatio-temporal prior can substantially expedite the overall identification problem.

## Background

Accurate plant species identification represents the basis for all aspects of plant related research and is an important component of workflows in plant ecological research [1]. Numerous activities, such as studying the biodiversity richness of a region, monitoring populations of endangered species, determining the impact of climate change on species distribution, and weed control actions depend on accurate identification skills. They are a necessity for physiologists, pharmacologists, conservation biologists, technical personnel of environmental agencies, or just fun for laypersons [2–4]. Expediting the task and making it feasible for non-experts is highly desirable, especially considering the continuous loss of plant biodiversity [5] as well as the continuous loss of plant taxonomists [6]. The principal challenge in plant identification arises from the vast number of potential species. Even when narrowing the focus to the flora of a single country, thousands of species need to be discriminated. The flora of Germany exhibits about 3800 indigenous species [7], the British & Irish flora comprises around 3000 [8], and the flora of Northern America exhibits about 20,000 species of vascular plants [9].

However, most species are not evenly distributed throughout a larger region as they require more or less specific combinations of biotic and abiotic factors and resources to be present for their development. Therefore, plant species can be encountered within their specific ranges. The German Biodiversity Exploratories project [10] studied sites spanning an area of 422 km^2^ to 1300 km^2^ and found that on grassland sites 318 to 365 vascular plant species occurred [11], while on forest sites merely 277 to 376 species were present [12]. These figures represent less than 10% of the entire German flora. Knowing where species occur has long been of interest, dating back to Linné and Humboldt with mapping projects evolving in terms of coverage and level of detail over time. A geographic range map represents the area throughout which a species occurs, referred to as ‘extent of occurrence’ by the *International Union for Conservation of Nature* (IUCN). Using range maps as they appear in field guides to support manual species identification has been state-of-the-art for quite some time. However, species identification is changing and the usability of field guides has often been debated. Taking a user’s current position in the field to estimate which species could possibly be encountered nearby can simplify identification tasks and is highly suitable given today’s prevalence of mobile devices with self-localization technology.

In this paper, we study whether previously recorded occurrence information can be used to develop a recommendation system to significantly reduce the number of species for the identification task. Resulting recommendations could either be used on their own or be incorporated into species identification services to improve accuracy [13]. We conduct a systematic study on different data sources and aggregation strategies to evaluate how accurately taxa can be retrieved depending on location and time of a new observation. We select the territory of Germany as study region since its flora is particularly well described with curated, openly available databases. In particular, we use the following two sources of data. First, grid-based range maps published by the *Federal Agency for Nature Conservation* via the FLORKART project. Second, plant observations obtained from the *Global Biodiversity Information Facility* (GBIF), a service aiming to mobilize biodiversity data from museums, surveys, and other data sources by collating locally digitized and stored data in an online data search portal [14].

Previous research exists in two different research directions: species distribution modeling as well as automated species and object identification.

### Species distribution modeling (SDM)

SDMs are associative models relating occurrence or abundance data of individual species at known locations to information on the environmental characteristics of those locations (modified from [15], [16]). Once trained, SDMs can predict suitable habitats for species based on the utilized environmental characteristics. While initial studies were mainly seeking insight into causal drivers of species distributions, recent studies focus on predicting distributions across landscapes to gain ecological and evolutionary insights that require extrapolation in space and time [15].

SDMs utilize occurrence data as answer set while training the model and identifying a characteristic set of predictor variables. This enables their application in areas that have not been intensively sampled or under hypothetically changing conditions, e.g., climate change. However, using a limited set of predictor variables often results in limited accuracy and spatial resolution. While these restrictions are acceptable for ecological and environmental research on larger scales, the problem we study requires spatially fine-grained estimations. Prediction results were found to strongly depend on sampling bias [17], sampling size [18, 19], and location uncertainty [20] decreasing the confidence in SDM results [21, 22]. Further challenges for SDMs include the improvement of methods for modeling presence-only data, model selection and evaluation as well as proper assessment of model uncertainty [23].

The *Map of Life* service uses SDM to provide certain species range maps for confined geographical areas. Different data sources such as expert species range maps, species occurrence records, and ecoregions, are aggregated to describe species distributions worldwide [24]. However, the service is hardly of any use for the purpose of species identification since for example the whole area of Germany seems to be discretized into ≈25 tiles and the only retrieved plant species for this region are ten conifer species.

The *Plant-O-Matic* app utilizes SDM to predict a list of all plant species expected to occur at a user’s location [25]. For its predictions, the approach uses a 100×100 *km* discretization grid and 3.6M observations of 89k non-cultivated plant species native in America. For rare species (30k) with only one or two observations the geographic range is defined as a 75,000 *km*^2^ square area surrounding the occurrence locations. For 12k species with three to four observations, the range is defined as convex hull enveloping all occurrence points. For the remaining 45k species with more than five occurrences, range maps were predicted using the MaxEnt SDM [26]. MaxEnt uses 19 layers of world climate data and 19 spatial filters capturing the geometry of the studied areas as predictor variables. The approach predicts rather long and non-ranked species lists given the coarse-grained computational discretization and the sparse observation data.

### Automated species and object identification

We found no study that utilizes the location of an observation to support the identification of unknown plant specimen despite intensive research and manifold studies in this area [27]. Previous studies largely focus on image recognition techniques for automated plant species identification [28], how those can be enhanced by careful selection of image types [29] and contextual information such as plant size [30]. However, there exists previous work on more general identification problems that utilizes location data.

Berg et al. used observation time and location of images for supporting automated bird species identification by computing spatio-temporal prior probabilities for the bird species’ occurrences in North America [31]. Bird-sighting records are discretized into spatio-temporal cubes of 1^′^ latitude-longitude and six days. The authors compute the prior at a given location and time as ratio of the estimated density of species observations and the estimated density of any observation at the same location and time. The authors used 75M bird-sighting records of 500 bird species originating from a citizen-science network. By combining image recognition and the spatio-temporal prior, top-5 accuracy of correctly identified bird specimen improved by 15% relatively (≈ 10% absolutely), indicating that the use of spatio-temporal priors can significantly support automated species identification.

Tang et al. studied the usage of location context for the problem of image classification for 100 location-sensitive classes such as ’Beach’, ’Disneyland’, and ’Mountain’ [32]. They constructed high-dimensional (>80k) feature vectors representing contextual information about images location. These features are computed per image location and derived from five sources: (1) a 25 ×25 km grid-based discretization of the location (20k dim); (2) normalized pixel colors from 17 ×17 px patches of ten map types referring to average vegetation, congressional district, ecoregions, elevation, hazardous waste, land cover, precipitation, solar resource, total energy, and wind resource (9k dim); (3) regional statistics on age, sex, race, family and relationships, income, health insurance, education, veteran status, disabilities, work status, and living conditions (21k dim); (4) hashtag frequency on Instagram at 10 radii (2k dim); (5) visual context as probability of 594 common concepts appearing on social media website at 10 radii (30k dim). Following a dimensional reduction, these context features are concatenated with the visual features and incorporated into a Convolutional Neural Network before its softmax layer. The authors report a 19% relative gain in mean average precision (7% absolute) and a 6% relative improvement of top-5 accuracy (4.5% absolute). Both studies clearly suggest that analyzing location and temporal context of an identification can substantially improve identification accuracy.

Our approach is unique in that it relies on actual observation data directly rather than inferring species distribution by means of a model taking these data as input for training. Being subject to model reliability and data quality issues [33], SDMs are used to predict a potential range whereas we base our estimation entirely on factual observations. Previous studies on automated species identification have shown the benefit of using location information for improving identification results. They did however not investigate the accuracy of ranked taxa recommendations retrieved directly from occurrence data. As such observation records are becoming increasingly available via online services, providing comprehensive sets of presence-absence as well as presence-only occurrence records, we argue that a systematic study is required that evaluates how spatio-temporal context information can be exploited to inform on-site plant species identification.

## Methods

### Study region and taxa

We use the territory of Germany as evaluation area for our study. Besides giving us the opportunity to test our estimations on site, Germany is representative for countries with well-documented species populations in range maps and specimen collections. Moreover, active groups of passionate professionals constantly contribute observation data [34].

In search of a complete species list, we decided to take the widely accepted list of ferns and vascular plants of Germany [35] collected by Wisskirchen and Haeupler [7] as a basis. The list was revised addressing the following two issues. First, some taxa are known to be exceptionally difficult to distinguish from each other, their identification relying on very special characters and often being impossible to accomplish in the field without a reference collection, even for experts. We subsumed 858 species belonging to five of these critical taxa [36] under their respective parent taxa *Ranunculus auricomus*, *Rubus*, *Sorbus*, *Taraxacum*, and *Hieracium*. Secondly, we excluded 251 hybrid species expected to cause inconsistent and unreliable identifications. Thus, our list is composed of 2,771 plant taxa containing 2,766 taxa at species level as well as four at genera and one at aggregate level being treated as leaves of the taxonomic scheme in our study.

### Grid-based presence-absence data

Grid-based presence-absence data stems from large-scale efforts to systematically map geographic regions. Being the most comprehensive data source for Germany and providing data for its entire area, we employ the FLORKART project. FLORKART is the result of cumulative mapping involving thousands of voluntary surveyors and literature reviews in several organizational subunits [37]. The data is freely accessible via the information system FloraWeb [38] run by the *Federal Agency for Nature Conservation* on behalf of the *German Network for Phytodiversity* (NetPhyD). In FLORKART, presence of a species is recorded on the basis of grid tiles, originally representing pages of ‘Messtischblatt’ (MTB) ordnance survey maps with a scale of 1:25,000. Each tile covers a section of 10’ longitude ×6’ latitude, corresponding to a surface area of approximately 118 *km*^2^ in the north to 140 *km*^2^ in the south of Germany. However, only 3.5% of FLORKART grid tiles are of this coarse-grained resolution, with many of them superseded. The majority of presence-absence information today is provided on the scale of quarter tiles, subdividing each MTB into four parts. In spite of the increased resolution each tile still only carries the binary information whether a species appears in it or not. Neither exact spatial coordinates of individual records nor frequency of a species’ occurrence are known.

FLORKART has proven to be of significant value for biogeographical analyses and the quality of its data has been validated in numerous studies, e.g., [39, 40].

**Fig. 1:**
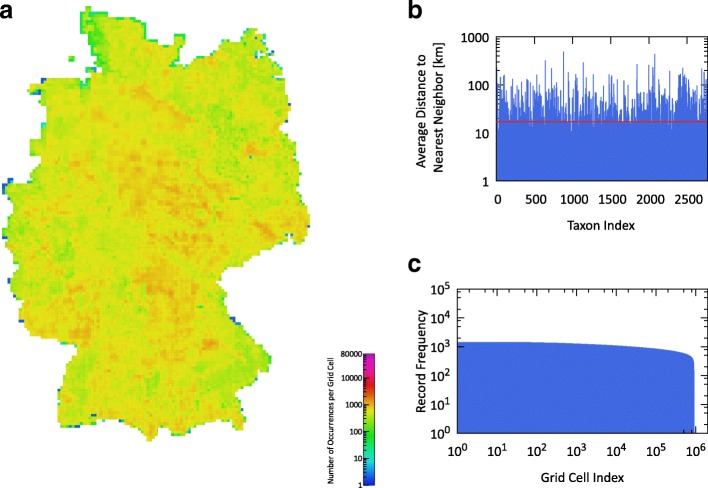
Characteristics of the FLORKART dataset – **a** spatial density of occurrence records per grid cell across all taxa; **b** average distance to nearest neighbor occurrence per taxon, average over all taxa marked by red line; **c** frequency distribution of occurrence records per grid cell

FLORKART contains records at all taxonomic levels, including subspecies and aggregates of species. For this study, records were revised in order to map them to our taxa list. In detail, records of child taxa, i.e., subspecies, forms and varieties of species, were included and subsumed under their respective parent taxon. In result, our FLORKART dataset contains presence-absence data for the 2771 vascular plant taxa in our species list. On May 3rd and 4th 2017, we acquired a total of 6.59M records for these taxa across the 13k (quarter-)MTB tiles entirely covering Germany. We discarded records that were marked as ’questionable’ or ’false’ (15k records). The remaining data were collected during three time periods: before 1950, between 1950 and 1980, and 1980 until today. In those cases where FLORKART provides records for a coarse-grained tile as well as for sub-quadrants within the same tile, we always consider the newer and higher-resolution information. This leads to a total of 6,020,296 records in our dataset, with only 0.54% of those accounting for coarse-grained tiles and 0.9% accounting for data from before 1950. A median of 514 taxa occurs per grid cell, with the 10th percentile being 257 and the 90th percentile being 758 taxa. Figure 1 displays the spatial density of the records mapped to the area of Germany as well as coverage metrics of the FLORKART dataset.

### Point-based occurrence records

We use the *Global Biodiversity Information Facility* (GBIF) as the most prominent and comprehensive data source for querying point-based occurrence records for Germany. Occurrence denotes one observation record of a certain plant and contains information on the taxonomic description, geographic location, observation type, and often also the observation time and date. The GBIF web service aggregates occurrence records of numerous types, from historic herbarium specimens to citizen science projects, e.g., hobbyists sharing geo-tagged species photos. The data differs considerably from the grid-based records described above in that it represents presence-only records being largely non-curated and collected unsystematically at arbitrary locations.

We queried GBIF via the website’s occurrences search interface, restricting records to the area of Germany and the biological kingdom of Plantae. All queries [41] were executed on August 23, 2017. The point-based occurrence records of interest for our study stem from 1324 datasets coming from 484 institutions with the largest contributor ’Naturgucker’ providing 27% of the records. We sanitized the data and filtered out invalid geographical locations, i.e., missing or implausible coordinates as well as entries with abnormally poor spatial accuracy. We mapped the taxa in our list to the GBIF taxonomic backbone using the ’species.search’ method of the GBIF API [42]. For every taxon, the query contained the accepted scientific name as well as synonyms, both including the author(s) describing the taxon. Approximate string matching was applied if the author naming was following a different convention, e.g., abbreviations.

**Fig. 2:**
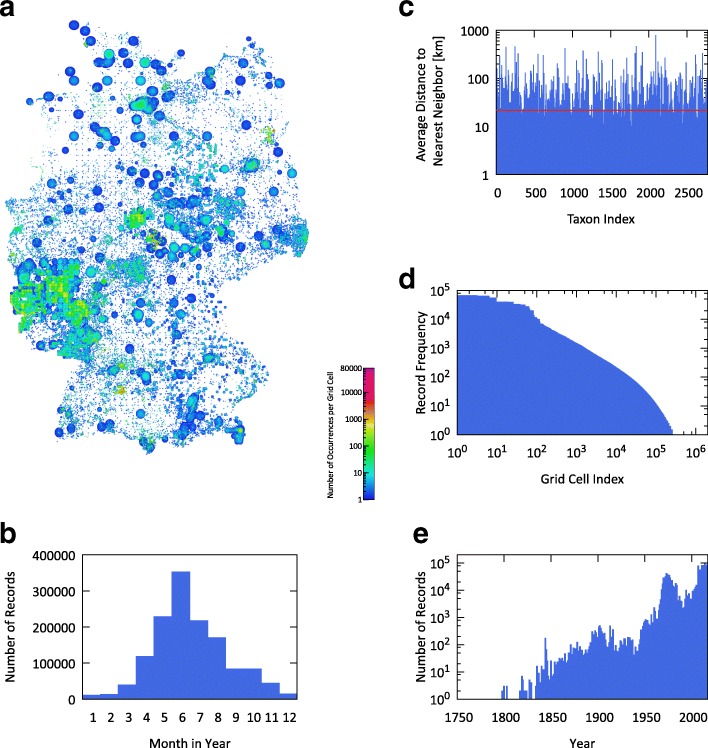
Characteristics of the GBIF dataset – **a** spatial density of occurrence records per grid cell across all taxa; **b** record distribution per month of observation; **c** average distance to nearest neighbor occurrence per taxon; **d** frequency distribution of occurrence records per grid cell; **e** record distribution per year of observation

In result, this process lead to a total of 1,598,550 occurrence records for 2,640 out of the 2771 taxa of interest in our study. The records contain a median number of 83 observations per taxon, with a 10th percentile of 4 and a 90th percentile of 1,817 observations per taxon. 86% of these records include plausible timestamps, e.g., they do not use default dates like January 1st 1970, and are distributed as visualized in Fig. 2(b) and (e). While single records date back to the year 1768 (i.e., herbarium specimen), 99% of the records with plausible timestamp are from 1950 and later.

In order to better understand how the retrieved GBIF records are distributed across Germany, we calculated per taxon the average distance between each observation and its closest neighbor (see Fig. 2(c)). Lower values indicate a spatial clustering of records, while higher values show dispersion of records. For comparison, we computed the same metric for the grid-based FLORKART data (see Fig. 1(b)). The average closest neighbor distance across all taxa in the GBIF dataset is 21.9 km, while the corresponding value is only 17.9 km for the FLORKART dataset. The figures and metrics illustrate the irregular distribution of records and gaps between records across the whole study region.

We discretized record locations into a regular computational grid with each cell spanning 30” longitude ×18” latitude. This discretization was chosen to provide a resolution 100 times higher than FLORKART’s quarter tiles and results in cells of ≈0.33 *km*^2^ each. We study the impact of the computational grid’s resolution in its own subsection below. Only 20% of the grid’s cells are occupied by GBIF records with a median of 4 occurrences, the 10th percentile being 1 and the 90th percentile being 56 records. The record frequency per occupied cell is heavily unbalanced with 50% of all occurrence records being concentrated in merely 0.8% of the occupied cells (cp. Fig. 2(d)). Figure 2(a) visualizes occurrences’ spatial density on a map of Germany with a circle depicting each record and its given accuracy and each colored pixel representing an computational grid cell. The map shows that even though records are sparse and irregularly distributed, they are spread across all parts of Germany. When classifying record locations in terms of land cover [43], 23% are on non-irrigated arable land, 16% on pastures, 15% in broad-leaved forests, 14% in coniferous forests, and 10% on discontinuous urban fabric.

### Independent test dataset

**Fig. 3:**
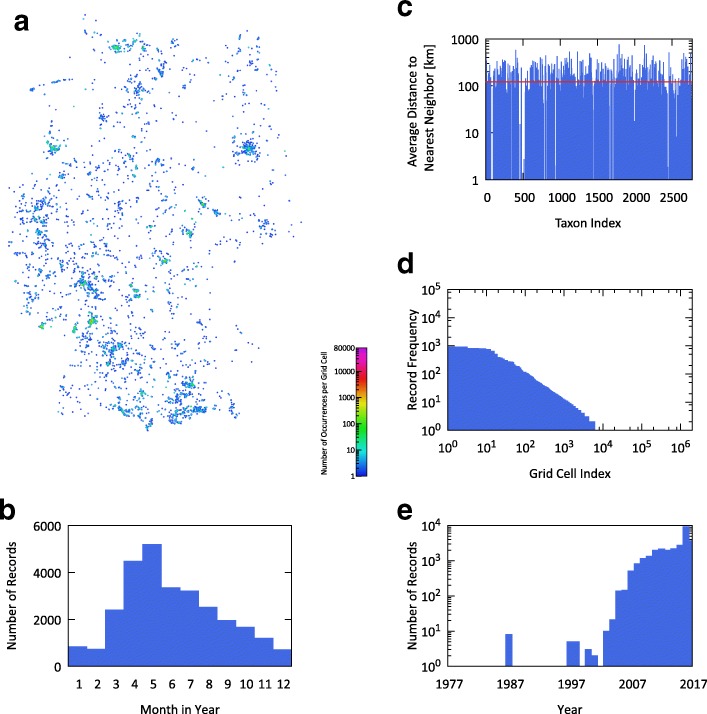
Characteristics of the Flickr test data – **a** spatial density of occurrence records per grid cell across all taxa; **b** record distribution per month of observation; **c** average distance to nearest neighbor occurrence per taxon; **d** frequency distribution of occurrence records per grid cell; **e** record distribution per year of observation

For obtaining an independent test set of occurrence data, we used the image hosting and social media website Flickr [44], a platform where users can upload and share personal photographs. We selected this service specifically because the uploaded images show what people actually ‘see’ and are interested in. We argue that this will to a large extent correlate with plant species people are interested in identifying and recording during their daily life. We used the Flickr API’s ’photos.search’ method to identify geotagged images labeled with the scientific name or an accepted synonym of the 2771 taxa considered in our study. From the images’ metadata we extracted the timestamp and the location of acquisition. This process resulted in 28,226 records for 1271 of the 2771 studied taxa. The summarized statistics are displayed in Fig. 3. In terms of geographical coverage across Germany, the test data is very sparse. Merely 0.69% of the computational grid cells as defined above are occupied having a median of 1 and a maximum of 1,127 records each. The number of records per occupied grid cell is biased, concentrated mainly around major urban areas and points of interest, but resembles that of GBIF (cp. Fig. 3(d) with Fig. 2(d)). Regarding land cover, most record locations (24%) are on discontinuous urban fabric, 19% on non-irrigated arable land, 12% on pastures, 9% in broad-leaved forests, and 9% in coniferous forests. Another indication of this dataset’s highly scattered geographical locations is given by the average nearest neighbor distances (see Fig. 3(c)) showing that data records exist on average only every 128.2 km. For a graphical overview of occurrences’ spatial density and the amount of geographical coverage see Fig. 3(a).

### Problem formalization and aggregation strategies

Given an observer’s location *p*∈*P* as geographic coordinates and date of observation *d*, we determine the candidate subset *T*_*p,d*_⊆*T* of all known taxa *T* that is most likely to be encountered by the observer. We hypothesize that spatial and temporal distance to registered occurrence records affect an observer’s chance to encounter the same taxa at their current location in the field. Therefore, we assign each taxon *t*∈*T*_*p,d*_ a score *S*_*t,p,d*_ reflecting its chance of being encountered at *p* and *d*.

$$ T_{p,d}=\left\{t_{i} \in T| S_{t_{i},p,d} > 0\right\} $$

The result will be a list of taxa, ranked based on scores. Hence, we denote a taxon’s rank by *r* and define the resulting ranked list of candidates $\widetilde {T}_{p,d}$ as:

$$\begin{array}{*{20}l} \widetilde{T}_{p,d}\!=&\left\{(t, r): t \in T_{p,d}, r \in \mathbb{N}:r \in [1,|T_{p,d}|],\right.\\ &\left.\forall t_{i}, t_{j}, r_{i}, r_{j}: (t_{i}, r_{i}) \in \widetilde{T}_{p,d} \land t_{i}=t_{j} \rightarrow (t_{j}, r_{j}) \notin \widetilde{T}_{p,d}\right\} \\ &\forall (t_{i},r_{i}), (t_{j},r_{j}) \in \widetilde{T}_{p,d}: S_{t_{i},p,d_{i}} \geq S_{t_{j},p,d_{j}} \rightarrow r_{i} < r_{j} \ . \end{array} $$

For our test region of Germany we study the quality of ranked candidate lists *T*_*p,d*_ by evaluating them based on the test data introduced above. Test records *n*=1…*N* are represented as a tuple containing the location *p*_*n*_, the observation date *d*_*n*_ and the labeled taxon *t*_*n*_. We let *T*_*p,d*_=*T*_*n*_ for all (*p*_*n*_,*d*_*n*_,*t*_*n*_) in our set of test records with *n* representing the index of the test query.

### Evaluation metrics

We aim to asses computed candidate subsets *T*_*n*_ in terms of completeness, compactness, and efficiency of the ranking and therefore introduce the following five metrics.

(1) Average recall *R* measures the ratio of correctly retrieved test records in relation to all test records and is computed as

1 $$ R = \frac{1}{N} \sum_{n=1}^{N} R_{n},\ \text{with}\ R_{n} = \left\{\begin{array}{cc} 1,& \quad\text{if}\, t_{n} \in T_{n}\\ 0,& \quad\text{if}\, t_{n} \notin T_{n} \end{array}\right.  $$

Average recall is not only computed for the whole retrieved list but also for subsets thereof, assessing completeness up to specific list positions. *R*_*k*_ refers to the average recall up to rank *k* and is computed by cutting off the list of results after the *k*-th position and calculating the average recall on the remaining sublist (cp. Eq. 1). We report *R*_*k*_ for *k*={20,514} with 20 items referring to a user-friendly shortlist of recommendations and 514 being the median number of taxa present per FLORKART grid tile, reflecting the average number of taxa occurring in a local region.

(2) Average list length *LL* measures the average number of retrieved candidate taxa across all *N* test records and is computed as

2 $$ {LL} = \frac{1}{N} \sum_{n=1}^{N} |T_{n}|.  $$

(3) Average list reduction *LR* measures across all *N* test records the number of retrieved candidate taxa in *T*_*n*_ in relation to the number of all known taxa *T*. We introduce this metric to better understand to what extent the identification problem can be simplified by reducing the number of potential taxa. Based on the total amount of taxa |*T*| and the number of taxa retrieved with the *n*th test query |*T*_*n*_|, *LR* is computed as

3 $$ {LR} = \frac{|T|}{N} \sum_{n=1}^{N} \frac{1}{|T_{n}|}.  $$

(4) Mean reciprocal rank *MRR* measures the ranking quality of retrieved candidate lists for a set of test records. The reciprocal rank is the multiplicative inverse of rank *r*_*n*_ of the correct taxon for the *n*th test query and *MRR* is the average of reciprocal ranks for the whole test set of *N* queries. A taxon’s reciprocal rank equals 0 if it is not on the retrieved list *T*_*n*_. MRR is computed as:

4 $$  {MRR} = \frac{1}{N} \sum_{n=1}^{N} \frac{1}{r_{n}}, \text{with} (t_{n},r_{n}) \in \widetilde{T}_{n}.  $$

(5) Median rank *M* measures the rank which at least half of selected taxa are ranked higher than and therefore provides an indication of the results’ compactness. Similar to *MRR*, it aims to judge the quality of the ranking and where in the ranked list the correct taxa appear after ranking. It is computed as

5 $$ {\begin{aligned} M\,=\,\min\left\{s \!\in\! \mathbb{N}\!:\! \sum_{r=1}^{s} \sum_{n=1}^{N}\left|(t_{n},r) \cap \widetilde{T}_{n}\right|\!\geq \! \frac{1}{2}\sum_{r=1}^{|T|} \sum_{n=1}^{N}\left|(t_{n},r) \!\cap\! \widetilde{T}_{n}\right|\right\} \end{aligned}}  $$

We define five strategies for aggregating multiple grid tiles and records per taxon depending on their spatial and temporal characteristics.

### Retrieval from grid-based presence-absence data

In a first set of experiments, we evaluate presence-absence data of the grid tile containing the test location *p*∈*P* and, depending on a variable radius parameter, also those in its vicinity to compute a set of candidate taxa at a given test location. Since it is not clear how accurate and up-to-date the available data is, we study how sampling within a circle around a test point with four increasing radii (1 km, 5 km, 10 km, and 20 km) in addition to sampling at the test point’s true location affects the quality of retrieved candidate taxa *T*_*p,d*_. The hypothesis being that taxa may extend their range over time and that in cases where a test point resides close to the border of a tile, its neighbor tile may be as relevant as the containing tile itself. We include additional tiles if their center location $\bar {p} \in \bar {P}$ falls within the sampling radius. The subset $\bar {P} \subseteq P$ contains tiles’ center locations only.

When considering an area rather than a single point, it may be necessary to aggregate presence records from multiple tiles. We select four distinct aggregation strategies to study their effect on the quality of retrieved candidate taxa *T*_*p,d*_. For each taxon *t*_*i*_∈*T*, we compute a score $S_{t_{i},p,d}$ based on one of these strategies and sort the list $\widetilde {T}_{ {p,d}}$ accordingly. These strategies either consider the relative frequency of a taxon’s occurrences within those grid tiles covered by the sampling circle of radius *r* or a normalized Euclidean distance *dist*(*p*_*a*_,*p*_*b*_) between the test point and eligible tiles’ centers defined as those falling within the sampling circle.

We let $P^{r}_{t_{i},p}$ denote the set of locations within radius *r* around *p* at which taxon *t*_*i*_ occurs

6 $$ P^{r}_{t_{i},p}=\left\{p_{i} \in \bar{P}\ |\ {counts}(t_{i}, p_{i}) > 0\ \land\ {dist}(p,p_{i}) \leq r\right\}.  $$

The function ${counts}:T \times P \to \mathbb {R}$ yields the number of taxon occurrences at a location p. The following four strategies S1 … S4 aggregate the individual contributions of occurrences in $P^{r}_{t_{i},p}$ in order to compute a rank for all *t*_*i*_∈*T*_*p*,*d*_.

- **S1** Relative frequency of occurrence records ranks taxa based on how often they occur within a radius of tiles being sampled:
  7 $$ S_{t_{i},p,d}=\frac{1}{|P^{r}_{t_{i},p}|}\sum_{p_{j} \in P^{r}_{t_{i},p}}{{counts}(t_{i}, p_{j})}.  $$
- **S2** Weighted relative frequency of occurrence records ranks taxa based on how often they occur within a radius with their proportion of contribution being reduced the farther away they occur from the center:
  8 $$ S_{t_{i},p,d}=\frac{1}{|P^{r}_{t_{i},p}|}\sum_{p_{j} \in P^{r}_{t_{i},p}}{\frac{1}{1+dist(p, p_{j})}{counts}(t_{i}, p_{j})}.  $$
- **S3** Minimum spatial distance to records’ tile centers ranks taxa within the sampling radius based on their closest spatial distance to the test location:
  9 $$ S_{t_{i},p,d}=1-\frac{\min_{p \in P^{r}_{t_{i},p}} dist(p, p_{j})} {\max_{p \in P^{r}_{t_{i},p}} dist(p, p_{j})}.  $$
- **S4** Average spatial distance to records’ tile centers ranks taxa within the sampling radius based on each taxon’s mean spatial distance to the test location:
  10 $$ S_{t_{i},p,d}=1-\frac{1}{|P^{r}_{t_{i},p}|}\frac{\sum_{p_{j} \in P^{r}_{t_{i},p}} dist(p, p_{j})} {\max_{p_{j} \in P^{r}_{t_{i},p}} dist(p, p_{j})}.  $$

In order to obtain the set of taxa *T*_*p,d*_, we query the grid tiles across all taxa at a test record’s location *p* and within a radius *r* for obtaining the taxa set *T*_*p,d*_.

### Retrieval from point-based taxon records

We evaluate estimation quality based on GBIF records using the same four aggregation strategies S1 … S4 that we studied for grid-based presence-absence data and additionally introduce a strategy S5, which considers temporal distance between the date of a test observation and point-based occurrence records.

- **S5** Temporal distance to months with recorded occurrences ranks taxa based on Gaussian-weighted average monthly score centered at the current/test record’s month:
  11 $$ \begin{aligned} S_{t_{i},p,d}&=\frac{1}{|P^{r}_{t_{i},p}|}\sum_{p_{j} \in P^{r}_{t_{i},p}} \sum_{m = 1}^{12}{{countsInMonth}}(t_{i}, p_{j}, m)\\ &\quad \times \frac{1}{{\sqrt {2\pi} }}e^{- \frac{1}{2} (m - month(d))^{2}}. \end{aligned}  $$

where the function $ {countsInMonth}: T \times P \times \mathbb {N} \to \mathbb {R}$ yields a taxon’s chance of occurring at a particular location during a particular month and $month: date \to \mathbb {N}$ provides the month of an observation date. S5 is only applicable for the 86% point-based occurrence records with valid timestamp. Considering the granularity in which blooming periods are usually specified, we discretize records observation date into either one or two out of twelve monthly bins proportionally to observation day’s distance to the middle of the month. We define the temporal distance between a test record’s month of year $m \in \mathbb {N}: m \in [1,12]$ and that taxa’s occurrences as the weighted sum of a taxon’s monthly scores having the maximal weight centered around the current month and decreasing both ways.

Although potentially being of high precision, GPS locations always suffer from certain spatial inaccuracies, often provided as an additional parameter along with the location. Over 35% of our GBIF records provide this additional value characterizing their spatial accuracy. For this reason and to mitigate the sparsity of GBIF point data, we consider each point of a recorded observation as having an influence on its surroundings. We treat coordinates of an occurrence record as center of a circle having a radius corresponding to its uncertainty with the expectation of a taxon’s encounter being highest at the center while linearly decreasing concentrically. For the remaining records without any indication of spatial accuracy we assume a default accuracy of 500 m reflecting the average accuracy of GBIF records providing this information in our study. Similar to the process described before, we query all point-based records within a radius *r* of a test record’s location *p* to sample occurrence frequencies and times for obtaining the taxa set *T*_*p,d*_.

### Retrieval from combined grid- and point-based data

**Fig. 4:**
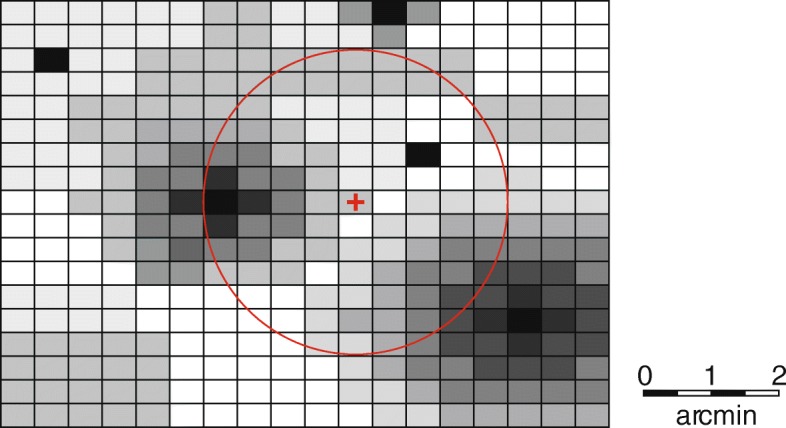
Grid section for a single taxon including area and point occurrences with different extents and uncertainties, respectively. The circle shows the sampling radius around the test position (red cross) being queried. The opacity of a tile is proportional to the taxon’s likelihood of being encountered there

In a final set of experiments, we investigate estimation quality based on merged grid-based presence-absence data and point-based taxa occurrence records. We apply the same five aggregation strategies S1 … S5 introduced above and are interested in understanding whether the combination of both data sources allows for a more complete and precise estimation of a taxon’s distribution. Figure 4 illustrates a possible configuration of a map segment aggregating both data sources for one taxon. Occurrence records with different accuracies as well as grid-based presence data at different scales contribute to an average value of how likely a taxon can be expected at a user’s location and its surroundings.

## Results

We assess the quality of taxa recommendations by measuring how accurately observations from the set of Flickr test data can be retrieved and report results of a series experiments on grid-based presence-absence data, point-based occurrence records, and a combination of both. In addition, we elaborate on how we run the experiments computationally efficiently. Metrics reported throughout this section include average recall (*R*), average list length (*LL*), average list reduction (*LR*), mean reciprocal rank (*MRR*) and median rank (*M*) as defined in the previous section.

### Ranked retrieval from grid-based presence-absence data

Table 1 summarizes the results of our first set of experiments retrieving ranked taxa lists from grid-based presence-absence data. From top to bottom, the table shows retrieval results at the exact location and for the four aggregation strategies S1 … S4. Per strategy we aggregate presence-absence data at four radii 1 km, 5 km, 10 km, and 20 km. The columns of the table refer to our previously introduced evaluation metrics.

**Table 1:** Results of ranked taxon retrieval solely using FLORKART grid-based presence-absence data sampled at the exact location and aggregated for increasing radii around Flickr test observations

Radius [km]	*R* [%]	*R*_20_ [%]	*R*_514_ [%]	*MRR* [%]	*M*	*LL*	*LR*
Retrieval at exact location							
0	82.31	3.38	64.13	1.11	307	680	4.54
S1: Relative frequency of occurrence records							
1	85.40	2.42	68.15	0.79	300	787	3.79
5	92.35	4.62	74.94	1.52	237	1115	2.59
10	94.47	4.78	74.42	1.35	234	1286	2.23
20	96.14	5.65	72.39	1.81	237	1477	1.92
S2: Weighted relative frequency of occurrence records							
1	85.40	2.62	68.73	0.95	287	787	3.79
5	92.35	4.36	74.80	1.56	240	1115	2.59
10	94.47	4.74	74.70	1.55	232	1286	2.23
20	96.14	5.71	73.88	1.78	233	1477	1.92
S3: Minimum spatial distance to records’ tile centers							
1	85.40	4.00	63.28	1.14	330	787	3.79
5	92.35	2.85	64.58	1.01	357	1115	2.59
10	94.47	2.13	64.25	0.80	375	1286	2.23
20	96.14	2.52	64.23	0.82	379	1477	1.92
S4: Average spatial distance to records’ tile centers							
1	85.40	2.06	60.00	0.65	380	787	3.79
5	92.35	0.46	52.91	0.37	470	1115	2.59
10	94.47	0.68	46.32	0.37	520	1286	2.23
20	96.14	0.81	37.00	0.37	615	1477	1.92

We observe a modest average recall of 82.31% when retrieving test observations from the grid cell at the exact position of a test record using solely presence-absence data. The recall increases up to 96.14% when aggregating data within radii of up to 20 km around a test location. *R* and *LR* depend only on the sampling radius and remain unaffected by the aggregation strategies S1 … S4.

While *R* is noticeably high meaning that an expected taxon likely appears somewhere on the retrieved list, its actual rank is rarely at the top as indicated by low *MRR* values. The same result is indicated by low median ranks, e.g., in merely half of the test cases the expected taxon ranks higher than 234th place using S1 and a radius of 10 km. In general, a higher recall of a larger sampling radius is achieved at the cost of an extended candidate list increasing from 680 taxa at the exact location to 1,477 taxa at a radius of 20 km (cp. Table 1). In consequence, we observe relatively poor ranking quality, illustrated by low values for *R*_20_ and median ranks >200 at all radii and across all aggregation strategies.

In terms of *MRR*, the methods relying on distances between test point and quadrant centers (S3 and S4) yield the poorest results. This can be attributed to a very small variety of unique distances, i.e., most taxa attaining the same score, which results from the comparatively coarse-grained FLORKART grid. The problem is less severe when relying on taxa frequency (S1 and S2). Since every FLORKART cell only documents the presence or absence of a particular taxon and not its frequency, these strategies are only applicable when the sampling radius spans multiple FLORKART cells. The weighted aggregation S2 additionally reduces the influence of records with increasing distance from the test location, which allows a finer gradation between center and neighborhood and thus more diverse score values. The effectiveness of this strategy is demonstrated by a 14.8% and 318.9% increase in *MRR* over S1 and S4 respectively as well as an improvement of the median rank *M* by 288 positions over S4 when sampling at a radius of 10 km.

### Ranked retrieval from point-based occurrence records

Table 2 summarizes the results of our second set of experiments on retrieving ranked taxa lists from point-based occurrence records. Overall, we observe considerably lower recall values compared to the previous set of experiments. At the exact location (r = 0 km), we achieve an average recall of 36.36%. However, with an increasing sampling radius this recall grows to 85.51% at r = 20 km.

**Table 2:** Results of ranked taxon retrieval solely using GBIF point-based occurrence records sampled at the exact location and aggregated for increasing radii around Flickr test observations

Radius [km]	*R* [%]	*R*_20_ [%]	*R*_514_ [%]	*MRR* [%]	*M*	*LL*	*LR*
S1: Relative frequency of occurrence records							
0	36.36	19.90	36.36	6.61	17	73	262.00
1	43.40	16.43	43.40	5.06	36	142	218.72
5	59.72	11.28	58.04	3.45	89	337	91.61
10	73.15	12.05	69.45	3.41	111	504	16.60
20	85.51	11.12	77.68	2.71	133	752	5.36
S2: Weighted relative frequency of occurrence records							
1	43.40	18.15	43.38	5.54	30	142	218.72
5	59.72	14.31	58.54	4.30	70	337	91.61
10	73.15	13.61	70.52	4.05	89	504	16.60
20	85.51	14.98	79.73	3.77	108	752	5.36
S3: Minimum spatial distance to records’ tile centers							
1	43.40	12.84	43.44	3.46	51	142	218.72
5	59.72	14.87	58.46	4.12	66	337	91.61
10	73.15	16.00	71.09	4.59	77	504	16.60
20	85.51	16.46	80.62	4.54	92	752	5.36
S4: Average spatial distance to records’ tile centers							
1	43.40	14.51	43.39	4.63	55	142	218.72
5	59.72	12.91	58.50	3.99	76	337	91.61
10	73.15	10.48	70.69	2.97	110	504	16.60
20	85.51	9.68	78.83	2.83	136	752	5.36
S5: Temporal distance to months with recorded occurrences							
0	36.35	23.12	36.35	7.36	13	73	261.10
1	43.39	19.81	43.39	5.81	24	141	218.97
5	59.71	12.47	58.84	3.60	77	337	91.78
10	73.15	11.21	69.95	3.08	108	503	16.67
20	85.50	7.25	77.88	1.96	168	751	5.37

We evaluated five ranking strategies for the retrieved taxa lists based on frequency, spatial distance, and temporal distance of occurrences. At a radius of 0 km, aggregation strategies S1 and S5 evaluate the exact computational grid cell of 0.33 km^2^ a test record falls into, producing highest *MRR* associated with lowest recall. The remaining strategies S2 … S4 consider spatial distance of records and can accordingly be applied only if the sampling radius spans multiple computational grid cells. Though yielding the same recall at respective radii, they differ in ranking quality as expressed by *MRR* and *M*. While S2 offers highest *MRR* up to 5 km, S3 improves for larger radii with results for S4 falling in between. Ranking based on temporal distance (S5) operates on the 86% GBIF records with an existing and valid observation time stamp alone. This reduced set of records explains the slightly differing figures in recall, list length, and list length reduction compared to S1. We found that *MRR* and median rank improve considerably when applying S5 making this strategy a promising option. Aggregating point-based records based on minimum spatial distance (S3) at a radius of 20 km was found to be the best performing strategy, yielding *R*=85.51*%*, *MRR* =4.54*%*, and *M*=92.

### Ranked retrieval from combined grid- and point-based data

Table 3 summarizes the results of our third set of experiments retrieving ranked taxa lists from a combination of grid-based presence-absence data and point-based occurrence records.

**Table 3:** Results of ranked taxon retrieval using FLORKART presence-absence data in combination with GBIF point-based occurrence records sampled at the exact location and aggregated for increasing radii around Flickr test observations

Radius [km]	*R* [%]	*R*_20_ [%]	*R*_514_ [%]	*MRR* [%]	*M*	*LL*	*LR*
S1: Relative frequency of occurrence records							
0	86.62	20.89	74.99	7.20	121	692	4.41
1	89.51	16.99	79.66	5.38	135	810	3.67
5	94.10	11.62	83.88	3.67	155	1142	2.54
10	95.98	12.19	83.03	3.55	160	1320	2.18
20	97.40	11.02	80.09	2.77	165	1525	1.86
S2: Weighted relative frequency of occurrence records							
1	89.51	19.67	80.35	6.00	116	810	3.67
5	94.10	15.38	84.33	4.60	131	1142	2.54
10	95.98	15.16	84.38	4.28	127	1320	2.18
20	97.40	15.00	84.08	3.83	128	1525	1.86
S3: Minimum spatial distance to records’ tile centers							
1	89.51	2.48	68.07	0.94	330	810	3.67
5	94.10	3.25	66.14	1.05	364	1142	2.54
10	95.98	1.90	65.72	0.84	378	1320	2.18
20	97.40	2.82	67.13	1.04	359	1525	1.86
S4: Average spatial distance to records’ tile centers							
1	89.51	3.70	63.18	1.81	374	810	3.67
5	94.10	1.09	52.77	0.66	478	1142	2.54
10	95.98	0.76	45.51	0.43	529	1320	2.18
20	97.40	1.05	36.70	0.42	624	1525	1.86
S5: Temporal distance to months with recorded occurrences							
0	36.35	23.15	36.35	7.37	13	73	261.10
1	43.39	19.86	43.39	5.76	25	141	218.97
5	59.71	12.52	58.82	3.60	77	337	91.78
10	73.15	11.06	69.95	3.04	108	503	16.67
20	85.50	7.22	77.87	1.98	167	751	5.37
S2+S5: Combined weighted relative frequency and temporal distance							
0	86.62	23.98	75.78	8.85	133	692	4.41
1	89.51	22.09	79.65	7.51	119	810	3.67
5	94.10	17.92	84.49	5.69	118	1142	2.54
10	95.98	18.14	85.25	5.12	112	1320	2.18
20	97.40	17.14	85.52	4.61	115	1525	1.86

The combination of both data sources increases recall in the computed candidate lists for all sampling radii, e.g., at *r*=20 km the individual recall of 96.14% (FLORKART) and 85.51% (GBIF) increase to 97.4% on the combined data.

Even more beneficial is the combination in terms of achieved ranking quality resulting in significantly improved results. Improvements are, for example, reflected in higher mean reciprocal rank (1.81% vs. 5.69%) and improved median rank (237 vs. 118) (cp. Table 1, S1 at 20 km with Table 3, S2+S5 at 5 km).

**Fig. 5:**
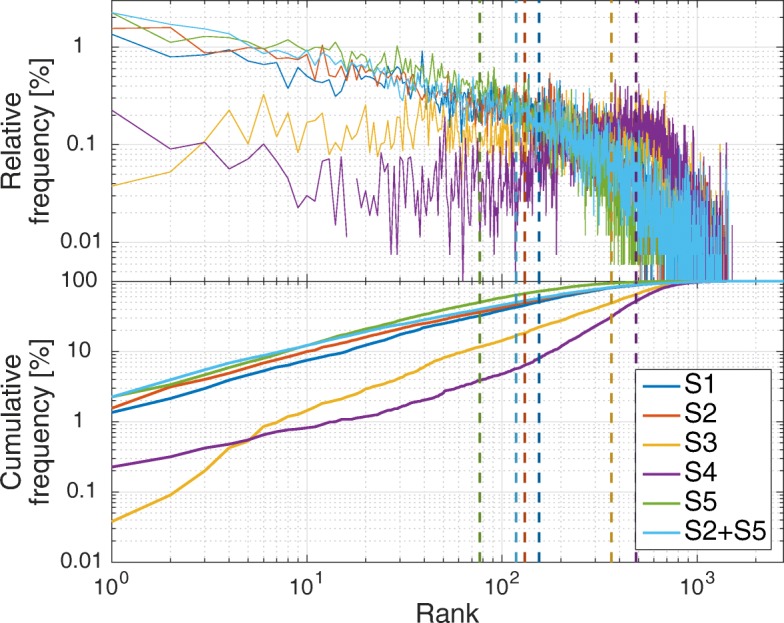
Relative and cumulative frequency per rank of correct taxon for recommending Flickr test records from FLORKART and GBIF datasets, using a search radius of 5 km and six different ranking strategies. The dashed vertical lines mark the median of each distribution

In addition to evaluating the scoring methods by themselves, we also studied linear combinations of those and found weighted spatial frequency with temporal scoring (see S2+S5 in Table 3) to yield the highest impact on *MRR* and *M*. For S2+S5, the 10th percentile rank is 521, the 90th percentile 8 and the median rank is 118. Figure 5 shows the distribution of ranks for the correct taxon per test record across all individual ranking strategies (S1 … S5) and the combination of spatio-temporal ranking (S2+S5) at an aggregation radius of 5 km. The figure shows that the correct taxon is ranked more frequently near the beginning of the list for S1, S2, S5, and S2+S5 and declining towards the end. The combination of S2+S5 shows additional benefits especially for the top ranks. S3 and S4 suffer from more evenly distributed frequencies over most ranks with a visible maximum around their respective median beyond the 350th rank.

We also wanted to assess the influence that a richer set of point-based occurrence records could have on our result. Therefore, we selected the three sites of the Biodiversity Exploratories project [10]: (a) *Schorfheide-Chorin*, (b) *Hainich-Dün* and (c) *Schwäbische Alb* as test cases. The sites span areas from 422 km^2^ to 1300 km^2^ and have been intensively investigated for plant species occurrences during systematic observations performed since 2006. The data is available on GBIF. However, our Flickr test observations proved to be very sparse for these regions with merely 13 records in the area of (a), 113 at (b), and 15 at (c). Given the very rich set of GBIF observations, we decided to perform a 10-fold cross-validation using 10% randomly selected GBIF occurrence records from the three areas (*N*_*a*_=76,696; *N*_*b*_=101,504; *N*_*c*_=104,968) as test set and only the remaining 90% as occurrence records. Table 4 reports results for the best performing aggregation method yet (S2+S5) and the combined taxa information consisting of presence-absence data and the 90% occurrence records. Each figure in the table is an average across the ten cross-validation runs. The results show that recall *R* as well as *R*_514_ are well above 99% in all three areas. High median ranks of 33 up to 17 and a *R*_20_ of 38% to 56% show the potential of predicting the sought-after taxon near the very top of a recommendation list.

**Table 4:** Results of ranked taxon retrieval in selected regions using combined using FLORKART areal data with 10-fold cross-validation on GBIF point data

Region	*R* [%]	*R*_20_ [%]	*R*_514_ [%]	*MRR* [%]	*M*	*LL*	*LR*
(a) Schorfheide-Chorin	99.95	56.39	99.86	17.42	17	943	2.95
(b) Hainich-Dün	99.72	48.16	99.59	13.08	22	1058	2.65
(c) Schwäbische Alb	99.95	38.03	99.83	10.47	33	935	2.98

### Considerations on computational efficiency

Apart from the influencing factors presented above, the quality of the taxa list depends on an actual implementation. One important consideration is the resolution of the computational grid used for binning occurrence records within close distance. A trade-off between required resources in terms of time and space and potential for improving evaluation metrics has to be made. We therefore varied the parameter of computational grid resolution while utilizing the best performing combined aggregation strategy S2+S5 with a sampling radius of *r*=10 km on joint FLORKART and GBIF data. Our implementation in C++ uses OpenMP to optimize for parallel execution where possible and was run on a state-of-the-art 10-core, 128GB RAM workstation. Resolution, expressed in relation to the quarter MTB tiles originally used to record presence-absence data, gradually increases from top to bottom in Table 5. Results show *R* remaining around 96%, while *R*_20_ and *R*_514_ increase slightly and the median rank improves up to 28 places at finer resolutions. We suspect that GBIF data is too sparse for a finer resolution to have a more pronounced impact. The discretization also introduces rounding errors which distort the results. Given the best tradeoff between *R* and *M*, we settled on a 0.33 km^2^ tile size being of 100 times finer granularity than FLORKART quarter tiles. This granularity provides the lowest median rank of 114 and an overall recall of 95.98%, it has been used for all other computations in this paper.

**Table 5:** Influence of grid resolution on evaluation metrics for S2+S5 and *r*=10 km

×Quarter	Avg. Area	Run-	RAM	*R*	*R*_20_	*R*_514_	*MRR*	*M*	*LL*	*LR*
MTB tile	[*km*^2^]	time	[GB]	[%]	[%]	[%]	[%]			
4	131.49	1.0 ×	0.5	96.45	16.14	84.00	4.92	140	1,349	2.12
1	32.87	1.1 ×	0.7	95.79	16.60	84.91	5.36	126	1,285	2.24
1/16	2.05	4.9 ×	5.7	96.20	17.85	85.13	5.36	114	1,331	2.16
1/64	0.51	15.4 ×	21.0	95.93	18.24	85.26	5.21	116	1,327	2.17
1/100	0.33	20.5 ×	33.2	95.98	18.19	85.24	5.14	112	1,320	2.18
1/144	0.23	29.6 ×	47.0	95.97	18.22	85.23	5.04	115	1,323	2.17

## Discussion

### Grid-based presence-absence data

Noticeably, recall does not reach 100% using grid-based FLORKART presence-absence data, but shows an increase when sampling a larger radius around the test location. While this may indicate that taxa extended their range since they were observed for FLORKART, it mainly suggests that our test data, being more representative of observations an interested hobbyist rather than a botanist may acquire in the field, are not accurately captured by FLORKART information alone. Flickr test records come from a multitude of users and also consist of cultivated plants observed in urban environments, e.g., city parks and (botany) gardens. Accordingly, the ten taxa most frequently failing correct prediction include ornamental and garden plants, such as *Narcissus pseudonarcissus* (Easter Lily), *Helleborus niger* (Christmas Rose), *Eranthis hyemalis* (Winter Aconite), *Helianthus annuus* (Common Sunflower), and *Leucanthemum vulgare* (Common Daisy) as well as cultivated and medicinal plants, such as *Brassica napus* (Rapeseed), *Cornus mas* (Cornelian Cherry), *Eschscholzia californica* (California poppy), and *Prunus cerasifera* (Cherry Plum). We should therefore seek to include taxa whose presence is not captured in wildlife presence-absence data. In addition to the mediocre retrieval performance, we also observe a relatively poor ranking quality as a direct result of using binary data without any notion of abundance. Using solely presence-absence data means that a rarely observed taxon will be ranked exactly the same as another, potentially very common one that occurs within the same grid tile.

### Point-based occurrence records

GBIF point-based occurrence records are spatially sparse and irregularly spread across the study region. Contrary to the presence-absence data, they have not been systematically sampled. Accordingly, we observe considerably lower average recall at the location of a test record. Using a larger sampling radius leads to substantially higher recall. At the largest evaluated radius of 20 km, we achieve a recall of 86% and an average candidate list length of 752 taxa. This list length is comparable to that computed based on the systematically sampled FLORKART data at comparable recall, i.e., 787 at 85%. This result raises expectations towards future use of GBIF data with its continuously increasing number of records. GBIF data offers an insight that presence-absence data do not provide. Multiple records of the same taxon in close proximity can be aggregated into an observation frequency allowing us to estimate which taxa a user would more likely try to identify. Using this information, we observe a substantially higher mean reciprocal rank and an improved median rank across all evaluated aggregation strategies S1 … S5. We found the minimum spatial distance S3 between a test record and existing GBIF records to yield the best ranking results.

### Combined grid- and point-based data

Occurrence records contributed to GBIF via citizen science projects are not limited to wildlife plant observations. Therefore, using both data sources in combination mitigates the missing predictions of taxa that are hard to estimate based on wildlife presence-absence data alone. We found that combining data sources yields the highest recall across all experiments with a maximum of 97.4% at a sampling radius of *r*=20 km. This result demonstrates that the different data sources are in fact complementary. Taxa that gain the largest absolute improvement by combining data are *Leucanthemum vulgare*, *Prunus cerasifera*, *Narcissus pseudonarcissus*, *Eranthis hyemalis*,*Cornus mas*, *Helleborus niger*, and *Brassica napus*. Although the recall improves by combining data, it still does not reach 100%, i.e., retrieved taxa lists are still incomplete with respect to the test observations obtained from Flickr. This is in part due to some locations and taxa which yield exceedingly low recall, i.e., false negatives when evaluated on the test data. False negatives dominantly occur at urban land cover types [43], i.e., discontinuous urban fabric (32%) and sport and leisure facilities (13%). Taking a closer look at the results of S2+S5 at *r*=5 *km*, the average recall is only 94.10% due to 345 individual taxa not being retrieved in the missing 5.90%. Among the top 66% of these 345 taxa, are 90.7% crop and garden plants. The top three are *Brassica napus*, *Narcissus pseudonarcissus*, and *Cornus mas*. These three taxa account for 13% of the missing recall alone.

In terms of candidate list ranking, we observed the best results by combining spatially weighted occurrence frequencies (S2) and temporal distance (S5) shown by consistently highest *MRR* values. Improved ranking allows for shorter candidate lists, which for instance is supported by *R*_514_ reaching a plateau around 84.5% at *r*=5 *km*, indicating a high chance of including the correct taxon before the 514th rank. An average list length of 1,142 at that distance shows that one would need to consider only 41% of all taxa of interest in Germany at a given location. Depending on the intended use case a compromise between recall and mean reciprocal rank has to be made. For a list as complete as possible one would consider a larger area to be sampled whereas a greater list length reduction can be achieved by sampling smaller regions.

An additional evaluation only at the three Biodiversity Exploratory sites yielded recall close to 100% and a remarkable 56% chance of the correct taxon being among the top 20 positions of a retrieved list. This result is very promising and shows how results can be improved with more point-based observation records in the future.

### Limitations

On average, our recommended list contains 1,142 taxa using a sampling radius of 5 km and S2+S5 strategy on combined observation data corresponding to a list reduction of 2.54. Despite being substantially reduced, the list is still long prompting us to understand whether the retrieved length is plausible. Studies [11, 12] recording species richness with respect to land cover found a total of 623 and 546 vascular plant species on grassland and forest plots, respectively. Since we do not consider land cover types for our study and base our estimations on FLORKART data with a maximal resolution of 30 *km*^2^ and a median number of 514 taxa per tile, we consider the resulting list lengths plausible. It is a future exercise to combine other data sources and to possibly increase resolution and precision of our estimations. To rule out the possibility of our own discretization having an adverse effect on data quality, we evaluated results across multiple resolutions as one aspect of our study.

Although being high, recall does not reach 100% in our experiments. One possible explanation is insufficient data quality since our datasets originate from manual acquisition processes. Revising maps with an extent such as FLORKART is an ongoing process that can never be expected to be complete. The range of species is highly dynamic as a consequence of, e.g., climatic differences and changes in land use. Some observations date back several decades while even the more current ones originate from mapping projects carried out in at least 47 federal project regions. GBIF’s observation records have been collected in an even more irregular manner, e.g., including citizen-science projects. We were able to mitigate some problems by analyzing data quality and eliminating erroneous records based on a set of heuristics (e.g., implausible dates and locations).

We purposely chose Flickr observations as test data since they reflect potential users and resemble a use case in which a taxon recommendation system could be applied. For instance, some test records are taken in urban environments (cp. Fig. 3), such as city parks, botany gardens and backyards. However, the data is neither curated nor verified by experts and is therefore expected to have errors, although verification of user-provided tags through image classification may yield improvements. Flickr records may be imprecise in the labeled taxa as well as the recorded location. In extreme cases, images may not be taken at the place of the original taxon occurrence, e.g., images of *Abies normannia* could show a Christmas tree in a living room. On the upside, this provides a chance of seeing results evaluated under a worst-case scenario. By conducting a cross-validation with GBIF records, we were able to show that our underlying method can yield results of much higher quality when operating on a richer and more fine-grained dataset.

## Conclusions

Recommending a list of plant taxa most likely to be observed at a given geographical location and time is useful for species identification as well as biodiversity research. We studied achievable recommendation quality based on two fundamental types of information, individually and in combination: binary presence-absence data and individually collected occurrence records. Furthermore, we aggregated data with increasing sampling radii around test locations and according to five formally defined aggregation strategies. Additionally, we investigated the influence of data discretization granularity on recommendation quality as well as on computational efficiency.

When relying solely on presence-absence data, the current state-of-the-art when looking for taxa that occur at a certain location, we managed to retrieve merely 82.31% of the test records, recommending the correct one at the 307th place in the list on average. By combining both data sources, increasing the sampling radius, and using a sophisticated aggregation strategy we were able to retrieve 95.98% of the test records, recommending the correct one on average at the 112th place in the list. When focusing on regions heavily sampled in terms of occurrence records, we even retrieved more than 99% of the test records’ taxa with the sought-after one ranking on average at the 24th place. In conclusion, we found that both studied data sources are highly complementary for use in a recommendation system. We demonstrated that such a system can be highly efficient in reducing the search space for species identification tasks with on average only 41% of all taxa needing to be considered at a given location. We also demonstrated that with the ongoing growth of species occurrence records in repositories like GBIF these results will constantly improve even further.
